# Supplementary material for: A Modular Metagenomics Pipeline Allowing for the Inclusion of Prior Knowledge Using the Example of Anaerobic Digestion
Source: Microorganisms. 2020 May 5;8(5):669. doi: 10.3390/microorganisms8050669 (PMC7284732; doi:10.3390/microorganisms8050669)
Supplement: Supplementary file 1 [file microorganisms-08-00669-s001.zip › supplementary flie 1.pdf]

Supplementary File 1

for

A Modular Metagenomics Pipeline Allowing for the Inclusion of Prior Knowledge Using the Example of Anaerobic Digestion

Daniela Becker, Denny Popp, Hauke Harms and Florian Centler *

Department of Environmental Microbiology, Helmholtz Centre for Environmental Research – UFZ, Permoserstraße 15, 04318 Leipzig, Germany; daniela.taraba@ufz.de (D.B.); denny.popp@ufz.de (D.P.); hauke.harms@ufz.de (H.H.)

* Correspondence: florian.centler@ufz.de

1. Evaluation of the performance of assembly and binning tools

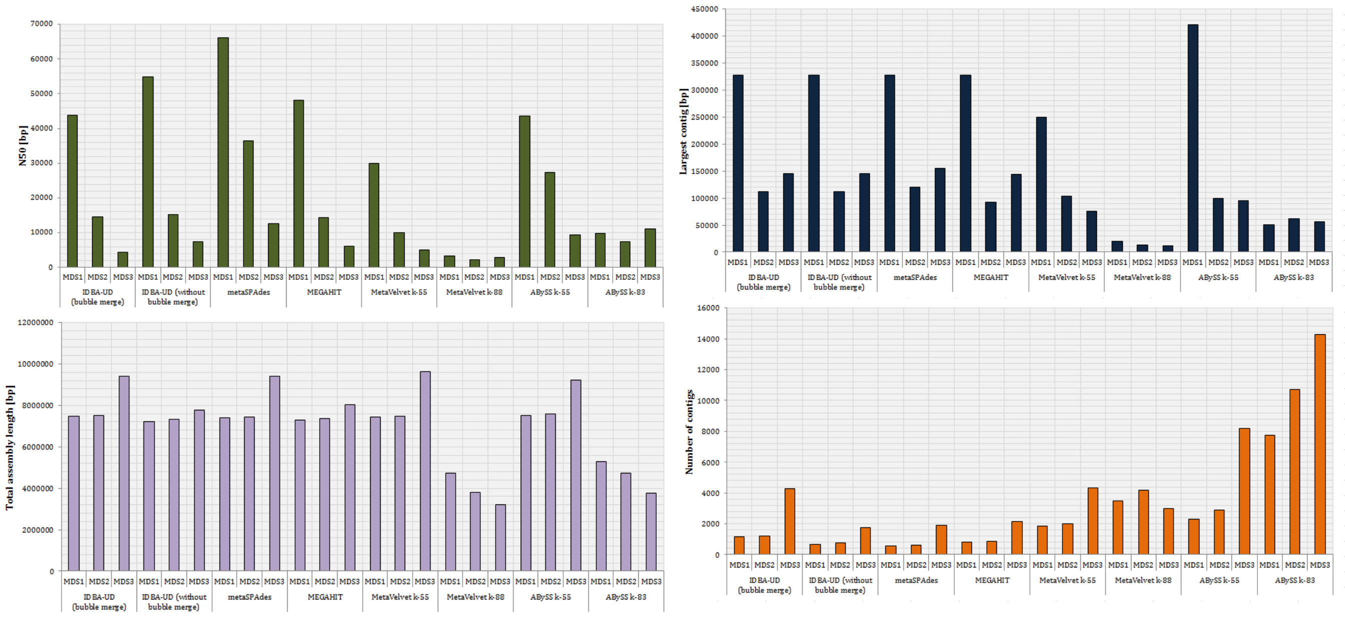

Figure S1: Quality statistics of assemblers per mock data set (MDS). ABySS [3,4] and MetaVelvet [5,6] are compared depending on two different k-mers. IDBA-UD [7,8], MEGAHIT [9], and metaSPAdes [10] were tested between a range of 51 bp and 201 bp on a step size of 10 bp. Input was total cleaned reads (paired and unpaired).

Table S1. Quality statistics of Maxbin2 performance (report based on CheckM).

Sample	Bin	Marker Lineage	Genomes	Marker	Completeness	Contamination	Strain Heterogeneity
**MDS1**	001	f__Enterobacteriaceae (UID5103)	157	1005	99.96	0.08	0
	002	p__Euryarchaeota (UID49)	95	228	95.32	2.29	40.00
**MDS2**	001	p__Euryarchaeota (UID49)	95	228	99.35	0.65	0
	002	f__Enterobacteriaceae (UID5103)	157	1005	99.08	3.26	82.14
**MDS3**	001	p__Euryarchaeota (UID49)	95	228	99.35	0.65	0
	002	p__Euryarchaeota (UID54)	90	234	96.51	3.94	70.00
	003	f__Enterobacteriaceae (UID5103)	157	1005	65.86	1.87	59.09

Table S2. Quality statistics of MetaBat performance (report based on CheckM).

Sample	Bin	Marker Lineage	Genomes	marker	Completeness	Contamination	Strain heterogeneity
**MDS1**	001	p__Euryarchaeota (UID49)	95	228	84.64	0.33	100.00
	002	f__Enterobacteriaceae (UID5103)	157	1005	99.96	0.08	0.00
**MDS2**	001	f__Enterobacteriaceae (UID5103)	157	1005	94.31	1.31	75.00
	002	p__Euryarchaeota (UID49)	95	228	99.35	0.65	0.00
**MDS3**	001	p__Euryarchaeota (UID49)	95	228	99.35	0.65	0.00
	002	p__Euryarchaeota (UID54)	90	234	90.28	1.60	75.00
	003	root (UID1)	5656	56	8.33	0.00	0.00

The results of IDBA-UD, metaSPAdes, and MEGAHIT are comparable (see Figure A.1).

2. Selected analysis strategies and assessing tool performance

To evaluate the benefit of including prior knowledge during data analysis, we compared results obtained by automated pipelines which require minor user input and intervention, and a custom-made pipeline which is carefully constructed by comparing tools for the different analysis steps. Most widely applied automated pipelines for taxonomic and functional analysis are the MG-RAST [1] web service and the interactive tool collection MEGAN6 [2].

Assembly evaluation

Popular assemblers using the de Bruijn graph algorithm were compared: ABySS [3,4] version 1.9.0, MetaVelvet [5,6] version 1.2.02, IDBA-UD [7,8] version 1.1.1, MEGAHIT [9] version 1.2.9, and metaSPAdes [10] version 3.14.0. The selection of assemblers follows the criteria given in Table 2 of the main manuscript. All assemblers were run with default parameter settings using the identical cleaned reads (paired and unpaired reads) of the MDSs as input. IDBA-UD and MEGAHIT were applied using pre-correction and metaSPAdes without. ABySS and MetaVelvet were run with two different k-mer settings. First, the k-mer value was calculated according to Zerbino (2010), taking the average read length (over all MDSs) minus 10 bp [11]. Second, an extended k-mer check covering the range from 51 bp (the default value) to 201 bp (ca. 80% of the maximal read length) and using a step size of 4 was performed for ABySS and MetaVelvet. In contrast, IDBA-UD, MEGAHIT, and metaSPAdes automatically examines k-mer values within a pre-defined range (51 to 201 bp with a step size 10) during the analysis. To adjust IDBA-UD for greater reads (> 128bp) and k-mers (>124) the scripts have to adjusted and the tool configured again (see A.4 section de novo analysis). The quality of the assemblies was assessed and compared based on N50, longest contig, contig amount, and total assembly length using QUAST [12] version 4.5.

Binning evaluation

As binning tools we tested MaxBin2 [13,14] version 2.2.3 and MetaBat [15] version 2.12.1 which are both two of the main popular binning tools and which both fulfil the criteria given in Table 2. MaxBin2 was run with the default optimization settings for the Expectation-Maximization (EM) algorithm with a minimum probability for EM algorithm of 0.8 (calculates the probability that a given scaffold belongs to any genome at the same time), with the additional experimental reassembly option and also with the marker set 40 as the default setting for communities which included mainly bacterial and archaeal species [11]. MetaBat was also used with the following default settings: sensitive mode (probability cutoff 90%, minimum probability for binning consideration 80%, minimum pearson correlation coefficient 92%, membership proportion 40% and proportion of shared memberships 20%), and unbinned option (created a folder with not binned contigs). As input for binning, we used the assembled reads (contigs) based on IDBA-UD assembly (here by using the merged-bubble option). Quality of derived bins was evaluated based on completeness and contamination calculated with CheckM [16] version 1.0.7.

Evaluation of automated pipelines

Cleaned reads and a meta-data file based on the MG-RAST template were uploaded per MDSs to the MG-RAST server. Default settings were chosen for the analysis.

As an alternative, cleaned reads were aligned with DIAMOND [17] version 0.8.36, mapped, and translated against an amino acid database (NCBI NR-database) and subsequently annotated using MEGAN6 [2] version 6.9.3 using eggNOG and protein accession of the NCBI-NR protein database. Applied databases were in detail: NCBI NR-database [26] for taxonomy, the eggNOG database [16,27] for functional information, and the protein accession of the NCBI-NR protein database based mapping [28]. Default parameter settings for short reads were chosen.

The best performing tools were combined to our custom-made pipeline. Analysis results based on our MDSs were compared for MG-RAST, MEGAN6, and our custom-made pipeline and compared to the known initial community composition. Additionally, we tested if including additional information in the custom-made pipeline had a beneficial effect.

3. Reference database

Table S3: Bacterial genomes which are included in the self-made database for pre-mapping

Species	Genome length [Mb]	Source
**Bacteria**		
Acetomicrobium hydrogeniformans	2.1	https://www.ncbi.nlm.nih.gov/genome/?term=Acetomicrobium+hydrogeniformans
Acetomicrobium mobile	2.2	https://www.ncbi.nlm.nih.gov/genome/?term=Acetomicrobium+mobile
Acholeplasma laidlawii	1.5	https://www.ncbi.nlm.nih.gov/genome/?term=Acholeplasma+laidlawii
Aminobacterium colombiense	2.0	https://www.ncbi.nlm.nih.gov/genome/?term=Aminobacterium+colombiense
Aminobacterium mobile	2.1	https://www.ncbi.nlm.nih.gov/genome/?term=Aminobacterium+mobile
Arcobacter anaerophilus	3.2	https://www.ncbi.nlm.nih.gov/genome/?term=Arcobacter+anaerophilus
Arcobacter cryaerophilus	2.1	https://www.ncbi.nlm.nih.gov/genome/?term=Arcobacter+cryaerophilus
Arcobacter faecis	2.5	https://www.ncbi.nlm.nih.gov/genome/?term=Arcobacter+faecis
Arcobacter lanthieri	2.3	https://www.ncbi.nlm.nih.gov/genome/?term=Arcobacter+lanthieri
Brachymonas chironomi	2.5	https://www.ncbi.nlm.nih.gov/genome/?term=Brachymonas+chironomi
Candidatus Cloacimonas acidaminovorans	2.2	https://www.ncbi.nlm.nih.gov/genome/?term=Candidatus+Cloacimonas+acidaminovorans
Comamonas aquatica	3.7	https://www.ncbi.nlm.nih.gov/genome/?term=Comamonas+aquatica
Comamonas composti	4.6	https://www.ncbi.nlm.nih.gov/genome/?term=Comamonas+composti
Comamonas granuli	3.5	https://www.ncbi.nlm.nih.gov/genome/?term=Comamonas+granuli
Comamonas testosteroni	5.6	https://www.ncbi.nlm.nih.gov/genome/?term=Comamonas+testosteroni
Desulfovibrio aespoeensis	3.6	https://www.ncbi.nlm.nih.gov/genome/?term=Desulfovibrio+aespoeensis
Desulfovibrio alcoholivorans	5.1	https://www.ncbi.nlm.nih.gov/genome/?term=Desulfovibrio+alcoholivorans
Desulfovibrio aminophilus	3.4	https://www.ncbi.nlm.nih.gov/genome/?term=Desulfovibrio+aminophilus
Desulfovibrio brasiliensis	3.6	https://www.ncbi.nlm.nih.gov/genome/?term=Desulfovibrio+brasiliensis
Desulfovibrio desulfuricans	3.3	https://www.ncbi.nlm.nih.gov/genome/15717
Desulfovibrio magneticus	4.6	https://www.ncbi.nlm.nih.gov/genome/?term=Desulfovibrio+magneticus
Desulfovibrio vulgaris	3.8	https://www.ncbi.nlm.nih.gov/genome/?term=Desulfovibrio+vulgaris
Mesotoga prima	2.3	https://www.ncbi.nlm.nih.gov/genome/?term=Mesotoga+prima
Pseudomonas fluorescens	6.3	https://www.ncbi.nlm.nih.gov/genome/?term=Pseudomonas+fluorescens
Pseudomonas putida	6.1	https://www.ncbi.nlm.nih.gov/genome/?term=Pseudomonas+putida
Syntrophaceticus schinkii	3.2	https://www.ncbi.nlm.nih.gov/genome/?term=Syntrophaceticus+schinkii
Syntrophobacter fumaroxidans	5.0	https://www.ncbi.nlm.nih.gov/genome/?term=Syntrophobacter+fumaroxidans
Syntrophomonas palmitatica	2.6	https://www.ncbi.nlm.nih.gov/genome/?term=Syntrophomonas+palmitatica
Syntrophomonas wolfei	2.9	https://www.ncbi.nlm.nih.gov/genome/?term=Syntrophomonas+wolfei
Syntrophomonas zehnderi	2.9	https://www.ncbi.nlm.nih.gov/genome/?term=Syntrophomonas+zehnderi
Thermanaerovibrio velox	1.89	https://www.ncbi.nlm.nih.gov/genome/?term=Thermanaerovibrio+velox
Thermovirga lienii	1.5	https://www.ncbi.nlm.nih.gov/genome/?term=Thermovirga+lienii

Table S4: Archaea genomes which are included in the self-made database for pre-mapping

Species	Genome Length [Mb]	Source
**Archaea**		
Methanobacterium arcticum	3.4	https://www.ncbi.nlm.nih.gov/genome/?term=Methanobacterium+arcticum
Methanobacterium congolense	2.5	https://www.ncbi.nlm.nih.gov/genome/?term=Methanobacterium+congolense
Methanobacterium formicicum	2.5	https://www.ncbi.nlm.nih.gov/genome/?term=Methanobacterium+formicicum
Methanobacterium lacus	2.6	https://www.ncbi.nlm.nih.gov/genome/?term=Methanobacterium+lacus
Methanobacterium veterum	3.4	https://www.ncbi.nlm.nih.gov/genome/?term=Methanobacterium+veterum
Methanococcus maripaludis	1.7	https://www.ncbi.nlm.nih.gov/genome/?term=Methanococcus+maripaludis
Methanococcus vannielii	1.7	https://www.ncbi.nlm.nih.gov/genome/?term=Methanococcus+vannielii
Methanococcus voltae	1.9	https://www.ncbi.nlm.nih.gov/genome/?term=Methanococcus+voltae
Methanoculleus chikugoensis	2.6	https://www.ncbi.nlm.nih.gov/genome/?term=Methanoculleus+chikugoensis
Methanoculleus horonobensis	2.4	https://www.ncbi.nlm.nih.gov/genome/?term=Methanoculleus+horonobensis
Methanoculleus marisnigri	2.3	https://www.ncbi.nlm.nih.gov/genome/?term=Methanoculleus+marisnigri
Methanoculleus sediminis	2.5	https://www.ncbi.nlm.nih.gov/genome/?term=Methanoculleus+sediminis
Methanoculleus thermophilus	2.2	https://www.ncbi.nlm.nih.gov/genome/?term=Methanoculleus+thermophilus
Methanomicrobium mobile	1.7	https://www.ncbi.nlm.nih.gov/genome/?term=Methanomicrobium+mobile
Methanoplanus limicola	3.2	https://www.ncbi.nlm.nih.gov/genome/?term=Methanoplanus+limicola
Methanoregula boonei	2.5	https://www.ncbi.nlm.nih.gov/genome/?term=Methanoregula+boonei
Methanoregula formicica	2.8	https://www.ncbi.nlm.nih.gov/genome/?term=Methanoregula+formicica
Methanosaeta concilii	3.0	https://www.ncbi.nlm.nih.gov/genome/?term=Methanosaeta+concilii
Methanosaeta harundinacea	2.3	https://www.ncbi.nlm.nih.gov/genome/?term=Methanosaeta+harundinacea
Methanosaeta thermophila	1.9	https://www.ncbi.nlm.nih.gov/genome/?term=Methanosaeta+thermophila
Methanosarcina acetivorans	5.8	https://www.ncbi.nlm.nih.gov/genome/?term=Methanosarcina+acetivorans
Methanosarcina barkeri	4.6	https://www.ncbi.nlm.nih.gov/genome/?term=Methanosarcina+barkeri
Methanosarcina horonobensis	5.0	https://www.ncbi.nlm.nih.gov/genome/?term=Methanosarcina+horonobensis
Methanosarcina mazei	4.1	https://www.ncbi.nlm.nih.gov/genome/?term=Methanosarcina+mazei
Methanosarcina siciliae	5.0	https://www.ncbi.nlm.nih.gov/genome/?term=Methanosarcina+siciliae
Methanosarcina thermophila	3.1	https://www.ncbi.nlm.nih.gov/genome/?term=Methanosarcina+thermophila
Methanosphaerula palustris	2.9	https://www.ncbi.nlm.nih.gov/genome/?term=Methanosphaerula+palustris
Methanospirillum hungatei	3.5	https://www.ncbi.nlm.nih.gov/genome/?term=Methanospirillum+hungatei

4. Commands and tool settings of the custom-made analyzing strategy

Pre-processing

First, raw reads per MDS were trimmed and quality filtered. Before and after raw as well as cleaned reads the quality was checked.

```
FastQC/fastqc org_input_forward.fastq –o out_directory/pre_check

FastQC/fastqc org_input_reverse.fastq –o out_directory/pre_check

java –jar Trimmomatic-0.36/trimmomatic-0.36.jar PE PE -phred33 org_input_forward.fastq org_input_reverse.fastq -baseout cleaned_output.fastq LEADING:30 TRAILING:30 SLIDINGWINDOW:4:30 AVGQUAL:30 MINLEN:30

FastQC/fastqc cleaned_input.fastq –o out_directory/post_check
```

Reads were automatically sorted by Trimmomatic [19] in 1P and 2P (1=forward and 2=reverse paired end reads) and 1U and 2U (1=forward and 2=reverse unpaired reads). If the header included spaces (like Illumina based header), the spaces were deleted to avoid information loss (e.g. forward reverse information per read) during analyzing by using

sed -e 's/ //g' before format transformation. After that, cleaned_output_1P.fastq and cleaned_output_2P.fastq were merged together to InterleavedPE_output.fastq by using shuffleSequences_fastq.pl (included in Velvet [6,11] version 1.2.0). Then fq2fa --merge –filter to generate InterleavedPE_output.fasta (included in IDBA-UD [7,8] version 1.1.1) were used. Additional cat was used to create a CombineUP_output.fastq (combined all unpaired reads) and Total_output.fastq file (unpaired and paired end reads combined).

Pre-Taxonomic-Scan

Prior knowledge about potentially included species (expected) could be useful to identify key abundant species to select expected species based reads. To reduce the data volume before *de novo* assembly the focus is only on unexpected species based reads. Metaxa2 [20] can be used to characterize first the expected species. Alternatively, information about community composition can be also gained by amplicon sequencing or fingerprinting methods as well as literature.

```
Metaxa2_2.1.3/metaxa2 -i Total_cleaned_input.fastq -o outdir/pretax -g SSU -q 20 -t bacteria,archaea

Metaxa2_2.1.3/metaxa2_ttt -i outdir/pretax.taxonomy.txt -o outdir/ttt_pretax -t bacteria,archaea --summary T --lists T --separate T --unknown T
```

After that, the whole genome sequences of identified organisms were downloaded from NCBI genome and compiled to small self-made reference database (see A.3) in form of a single multi-line fasta file per species.

Selection of aligned (expected) and unaligned (unexpected) reads

All reference genomes based on pre-taxonomic-scan or known microbiomes from literature research were indexed in one step using bowtie2-build as comma separated input. After that, cleaned reads were mapped against the indexed self-made reference database using Bowtie2 [21].

```
bowtie2-2.3.2/bowtie2-build referencegenome1,…,referencegenomeN idx_dir/index

bowtie2-2.3.2/bowtie2-inspect -s idx_dir/index 2>&1 | tee -a idx_inspect_logfile.txt

bowtie2-2.3.2/./bowtie2 --sensitive-local -a --fr -x idx_dir/index -1 Clean_input_1P.fastq -2 Clean_input_2P.fastq -U  CombineUP_output.fastq -S outdir/alignment.sam

samtools view -bS outdir/alignment.sam > outdir/alignment.bam

samtools sort outdir/alignment.bam -o outdir/alignment.sorted.bam

samtools index outdir/alignment.sorted.bam

samtools idxstats outdir/alignment.sorted.bam > outdir/idx_stats.txt
```

Subsequently, the alignment which included the aligned (expected) and separate the unaligned (unexpected) reads was extracted using SAMtools [22,23] flags.

```
samtools view -u -f 12 outdir/alignment.bam > outdir/interleaved_unaligned.bam

samtools view -u -f 4 outdir/alignment.bam > outdir/total_unaligned.bam

samtools view -u -F 2316 outdir/alignment.bam > outdir/total_aligned.bam
```

It could be possible that the generated BAM files were fixed and sorted again after selection also with SAMtools. After all, the BAM files were transformed in fastq files using BAMtools [24] and further in fasta format using FastX-Toolkit [29].

De novo analysis

The *de novo* analysis part combines *de novo* assembly, binning and reassembly. To avoid the duplication of sequence parts here the –no_bubble option was chosen. Alternatively, this option can be replaced by –merge_bubble. Thereby the complexity of the graph will be simplified. For more information see "Meta-IDBA: a de Novo assembler for metagenomic data" by Peng et al. 2011 [25]. One short note: IDBA-UD was by default defined for short reads until length of 128 bp and k-mers 124. To transform these value for greater reads the script short_sequence.h (1) and kmer.h (2) must be redefined as follows, if you like to use for example reads up to 312 bp length and maximal k-mers

of 312, modify the following values: (1) kMaxShortSequence=312 and (2) kNumUnit64=10 (corresponded to k-mer 312). The values can be modified individually.

```
MaxBin-2.2.3/auxiliary/idba-1.1.1/bin/./idba_ud -r outdir/interleaved_unaligned.fasta -o outdir/assembly –mink 51 –maxk 201 --step 10 –min_contig 1000 –no_bubble --pre_correction –num_threads 2
```

```
quast-4.5/./quast.py outdir/assembly1/contig.fa outdir/assembly2/contig.fa outdir/assembly3/contig.fa -o outdir/quast

MaxBin-2.2.3/./run_MaxBin.pl -contig outdir/assembly/contig.fa -reads interleaved_unaligned.fasta -reassembly -prob_threshold 0.8 -plotmarker -markerset 40 -out outdir/Bin -thread 1

checkm lineage_wf -r -x fasta –nt -t 1 --reduced_tree –pplacer_threads 1 outdir/Bin/ outdir/CheckM >> outdir/CheckM/logfile
```

Reassembly was performed for every generated bin separately (number of bin $B) and all reassembled contigs were bring together. Before, the header was transformed, depending on sample ID and bin number ($B) to provide the possibility to distinguish the bins.

```
MaxBin-2.2.3/auxiliary/idba-1.1.1/bin/./idba_ud -r outdir/Bin.reassem/Bin.reads.$B -o outdir/reassembly_$B –mink 51 --maxk 201 –step 10 –min_contig 1000 –no_bubble –pre_correction –num_threads 2

sed s/\>contig/\>contig_SampleID_$B /g outdir/reassembly_$B /contig.fa > contig_${meinarrayNEW[$i]}_bin_$B.fa

cp contig_${meinarrayNEW[$i]}_bin_$B.fa outdir/RAcontigs/

cat outdir/RAcontigs/*.fa > outdir/RAcontigs/total_contigs.fasta
```

Relative abundances were calculated based on the number of reads per contigs of the unexpected read reassembly in order to combine finally both information of expected read and unexpected contig annotation per MDS (whole community analysis). Therefore the unexpected reads were mapped against the unexpected reassembled contigs. The final stats_READSperCONTIG.txt files included the contig-name, contig length, read count, and in the last column the unmapped value (should be 0). This stats*.txt file was the basis for the read count calculation (see A.5)

```
bowtie2-2.3.2/./bowtie2-build outdir/RAcontigs/total_contigs.fasta outdir/RAcontigs/total_contig_idx
```

```
bowtie2-2.3.2/./bowtie2 -a -x outdir/RAcontigs/total_contig_idx -f outdir/total_unaligned.fasta -S outdir/readsTOcontigs.sam
```

```
samtools view -bS outdir/readsTOcontigs.sam > outdir/readsTOcontigs.bam

samtools sort outdir/readsTOcontigs.bam -o outdir/readsTOcontigs.sorted.bam

samtools index outdir/readsTOcontigs.sorted.bam

samtools idxstats outdir/readsTOcontigs.sorted.bam > outdir/stats_READSperCONTIG.txt
```

Taxa and functional annotation

Because DIAMOND mapping and MEGAN6 annotation has a large memory requirement (around 100 GB) and long run time (depending on data size, here more than 1 day) they should be run on a cluster system. NCBI NR-database version 2017-03-27 available under: ftp://ftp.ncbi.nih.gov/blast/db/FASTA/nr.gz (last access November 2017) was used.

```
diamond makedb –in nr.gz –d nr
```

```
approach of unexpected contig (long reads):

diamond blastx -q outdir/RAcontigs/total_contigs.fasta -d nr -a outdir/RAcontigs/total_contigs.daa -e 0.001 –matrix BLOSUM62 -b2.0

megan/./daa2rma –i outdir/RAcontigs/total_contigs.daa -o outdir/RAcontigs/total_contigs.rma –lg true -ms 50.0 -me 0.01 -alg naive -ram readCount -a2t prot_acc2tax-May2017.abin -a2eggnog acc2eggnog-Oct2016X.abin -fun EGGNOG

approach of expected reads:

diamond blastx -q outdir/total_aligned.fasta -d nr -a outdir/total_aligned.daa -e 0.001 –matrix BLOSUM62 -b2.0

megan/./daa2rma –i outdir/total_aligned.daa -o outdir/total_aligned.rma -ms 50.0 -me 0.01 -alg naive -ram readCount -a2t prot_acc2tax-May2017.abin -a2eggnog acc2eggnog-Oct2016X.abin -fun EGGNOG
```

Finally, the rma file was loaded into the MEGAN6 GUI. Calculation of the potential community is stored in Supplementary Material B.

Note: To reduce the run time of diamond the number of threads could be increased. Additional to avoid the generation of a new data format (rma), it is also possible to "meganize" the daa files directly. To avoid defect data copy original daa file after the BLASTX run should by stored as backup.

5. Additional statistical analysis using R

5.1 Calculate read counts per contig

In order to calculate the read counts per contig, the reads based on unexpected species must be mapped against the contigs using Bowtie2 (see A.4).

(1) Use the idxstats function of SAMtools to generate the summarized file about contig name, contig length and number of reads.
(2) Select the taxa level of interest and export "readName_to_taxonName" in CSV format by using MEGAN6.
(3) Third, the following R script could be used to combine these both information like following:

```
mapedreadstocontigs <- "idx_stats.txt"
contigtaxaname <- "readname_to_taxonname.txt"

###read the input and transform the table header
CountTab <- read.table(file=mapedreadstocontigs, sep="\t", quote="\"")
TaxTab <- read.table(file=contigtaxaname, sep="\t", quote="\"")
colnames(CountTab) <- c("contigname", "length", "readcount","unmapped")
colnames(TaxTab) <- c("contigname", "taxa")

###merge tables
readcountsALL <- merge(CountTab, TaxTab, all=TRUE)
# extract the SampleID as a separate data column from the contig name
readcountsALL$SampleID <- gsub("_[^_]*_[^_]*$", "", readcountsALL$contigname)
# count readcount entries over unique taxa - SampleID combinations
myTable <- xtabs(readcount~taxa+SampleID, readcountsALL)
write.table(readcountsALL, "readcount-per-contig-taxonname.txt", sep="\t")
write.table(myTable, "sum_readcount-per-contig-taxonname.txt", sep="\t")
```

5.2 Combine taxonomic and functional information

A similar functional potential between different MDSs was detected. Therefore, the combination of taxonomy and function was investigated to analyze changes depending on compositional differences.

Use MEGAN6 to export the necessary information.

(1) Extract the taxonomy information by uncollapsing all nodes and select all nodes of the taxonomy tree.
(2) Then, the data via the option "readName_to_taxonPathPercent" (summarized or assigned and tabulator separated) must be exported.
(3) The functional information (here EggNOG) by uncollapsing all nodes of the functional tree was selected and only the last level (all leaves) was highlighted.
(4) Finally, the "readName_to_eggnogPath" in tabulator separated format as CSV was exported.
(5) Then, the following R script was used:

```
# Assuming unique info for each tax and func! Check this in input data!
```

```
taxFile <- " SampleID-taxa.txt"
funcFile <- "SampleID-fct.txt"

# Get read ids as row headers, taxonomic info as column V2
taxData <- read.table(file=taxFile, sep="\t", row.names=1, quote="\"")

# Parse taxonomic info and put in columns
taxData$Kingdom <- gsub(pattern=";.*", replacement="", gsub(pattern="^.*d__", replacement="", taxData$V2))
taxData$Phylum <- gsub(pattern=";.*", replacement="", gsub(pattern="^.*p__", replacement="", taxData$V2))
taxData$Class <- gsub(pattern=";.*", replacement="", gsub(pattern="^.*c__", replacement="", taxData$V2))
taxData$Order <- gsub(pattern=";.*", replacement="", gsub(pattern="^.*o__", replacement="", taxData$V2))
taxData$Family <- gsub(pattern=";.*", replacement="", gsub(pattern="^.*f__", replacement="", taxData$V2))
taxData$Genus <- gsub(pattern=";.*", replacement="", gsub(pattern="^.*g__", replacement="", taxData$V2))
taxData$Species <- gsub(pattern=";.*", replacement="", gsub(pattern="^.*s__", replacement="", taxData$V2))
taxData$taxInfo <- TRUE # mark read to have taxonomic info

# Record percentage info

taxData$KingdomPercent <- gsub(pattern=";.*", replacement="", gsub(pattern="^.*d__[^;]+;", replacement="", taxData$V2))
taxData$PhylumPercent <- gsub(pattern=";.*", replacement="", gsub(pattern="^.*p__[^;]+;", replacement="", taxData$V2))
taxData$ClassPercent <- gsub(pattern=";.*", replacement="", gsub(pattern="^.*c__[^;]+;", replacement="", taxData$V2))
taxData$OrderPercent <- gsub(pattern=";.*", replacement="", gsub(pattern="^.*o__[^;]+;", replacement="", taxData$V2))
taxData$FamilyPercent <- gsub(pattern=";.*", replacement="", gsub(pattern="^.*f__[^;]+;", replacement="", taxData$V2))
taxData$GenusPercent <- gsub(pattern=";.*", replacement="", gsub(pattern="^.*g__[^;]+;", replacement="", taxData$V2))
taxData$SpeciesPercent <- gsub(pattern=";.*", replacement="", gsub(pattern="^.*s__[^;]+;", replacement="", taxData$V2))

# drop parsed taxonomic input string
taxData$V2 <- NULL

# put NA to entries which were missing
for (i in 1:7) {
  taxData[grepl('__', taxData[,i]), i] <- NA
}
for (i in 9:15) {
  taxData[grepl('__', taxData[,i]), i] <- NA
}

# get functional info
funcData <- read.table(file=funcFile, sep="\t", row.names=1, quote="\"")
funcData$Level1 <- gsub(pattern=";.*", replacement="", gsub(pattern="^[^;]*;", replacement="", funcData$V2))
funcData$Level2 <- gsub(pattern=";.*", replacement="", gsub(pattern="^[^;]*;[^;]*;", replacement="", funcData$V2))
funcData$Level3 <- gsub(pattern=";.*", replacement="", gsub(pattern="^[^;]*;[^;]*;[^;]*;", replacement="", funcData$V2))
funcData$Level4 <- gsub(pattern=";.*", replacement="", gsub(pattern="^[^;]*;[^;]*;[^;]*;[^;]*;", replacement="", funcData$V2))
funcData$Level5 <- gsub(pattern=";.*", replacement="", gsub(pattern="^[^;]*;[^;]*;[^;]*;[^;]*;[^;]*;", replacement="", funcData$V2))
funcData$Level6 <- gsub(pattern=";.*", replacement="", gsub(pattern="^[^;]*;[^;]*;[^;]*;[^;]*;[^;]*;[^;]*;", replacement="", funcData$V2))
funcData$funcInfo <- TRUE # mark read to have functional info

# put NA to entries which were missing
funcData[funcData==" eggNOG"] <- ""
```

```
funcData[funcData==""] <- NA

# drop parsed taxonomic input string
funcData$V2 <- NULL

# if only reads are of interest which have both taxonomic info and functional info use:
data <- merge(taxData, funcData, by = "row.names")

# lets get a table combining the levels of interest
# Select the taxa and eggnog level of interest (here exemplary: Species and EggNOG Level1)
myTable <- table(data$Species, data$Level1, useNA="always")
write.table(myTable, "myTable_sampleID_taxa-vs-fct.txt", sep = "\t")
```

References

1. Meyer F, Paarmann D, D'Souza M, Olson R, Glass EM, Kubal M, et al. The metagenomics RAST server – a public resource for the automatic phylogenetic and functional analysis of metagenomes. BMC Bioinformatics [Internet]. BioMed Central; 2008 Sep 19;9:386. Available from: http://www.ncbi.nlm.nih.gov/pmc/articles/PMC2563014/

2. Huson DH, Beier S, Flade I, Górska A, El-hadidi M. MEGAN Community Edition - Interactive Exploration and Analysis of Large-Scale Microbiome Sequencing Data. 2016;1–12.

3. Simpson JT, Wong K, Jackman SD, Schein JE, Jones SJM. ABySS : A parallel assembler for short read sequence data. 2009;1117–23.

4. Jackman SD, Yeo S, Coombe L, Warren RL. ABySS 2 . 0 : Resource-Efficient Assembly of Large Genomes using a Bloom Filter Effect of Bloom Filter. 2017;768–77.

5. Namiki T, Hachiya T, Tanaka H, Sakakibara Y. MetaVelvet: An extension of Velvet assembler to de novo metagenome assembly from short sequence reads. Nucleic Acids Res. 2012;40(20).

6. Zerbino DR, Birney E. Velvet: Algorithms for de novo short read assembly using de Bruijn graphs. Genome Res. 2008;18(5):821–9.

7. Peng Y, Leung HCM, Yiu SM, Chin FYL. IDBA-UD: A de novo assembler for single-cell and metagenomic sequencing data with highly uneven depth. Bioinformatics. 2012;28(11):1420–8.

8. Peng Y, Leung H, Yiu S, Chin F. IDBA - A practical iterative De Bruijn graph De Novo assembler. Vol. 6044, Research in Computational Molecular Biology. 2010. 426–440 p.

9. Li D, Liu CM, Luo R, Sadakane K, Lam TW. MEGAHIT: An ultra-fast single-node solution for large and complex metagenomics assembly via succinct de Bruijn graph. Bioinformatics. 2015;31(10).

10. Nurk S, Meleshko D, Korobeynikov A, Pevzner PA. MetaSPAdes: A new versatile metagenomic assembler. Genome Res. 2017;27(5):824–34.

11. Zerbino DR. Using the Velvet de novo assembler for short-read sequencing technologies. Curr Protoc Bioinformatics [Internet]. 2010 Sep;CHAPTER:Unit-11.5. Available from: http://www.ncbi.nlm.nih.gov/pmc/articles/PMC2952100/

12. Gurevich A, Saveliev V, Vyahhi N, Tesler G. QUAST: quality assessment tool for genome assemblies. Bioinformatics [Internet]. 2013 Apr 15;29(8):1072–5. Available from: http://dx.doi.org/10.1093/bioinformatics/btt086

13. Wu Y-W, Tang Y-H, Tringe S, Simmons B, Singer S. MaxBin: An automated binning method to recover individual genomes from metagenomes using an expectation-maximization algorithm. Vol. 2, Microbiome.

2014. 26 p.

14. Wu Y-W, Simmons B, Singer S. MaxBin 2.0: An automated binning algorithm to recover genomes from multiple metagenomic datasets. Vol. 32, Bioinformatics (Oxford, England). 2015.

15. Kang DD, Froula J, Egan R, Wang Z. MetaBAT, an efficient tool for accurately reconstructing single genomes from complex microbial communities. Rahmann S, editor. PeerJ [Internet]. San Francisco, USA: PeerJ Inc.; 2015 Apr 2;3:e1165. Available from: http://www.ncbi.nlm.nih.gov/pmc/articles/PMC4556158/

16. Parks DH, Imelfort M, Skennerton CT, Hugenholtz P, Tyson GW. CheckM: assessing the quality of microbial genomes recovered from. Cold Spring Harb Lab Press Method. 2015;1–31.

17. Buchfink B, Xie C, Huson D. Fast and sensitive protein alignment using DIAMOND. Vol. 12, Nature methods. 2014.

18. Huerta-Cepas J, Szklarczyk D, Forslund K, Cook H, Heller D, Walter MC, et al. EGGNOG 4.5: A hierarchical orthology framework with improved functional annotations for eukaryotic, prokaryotic and viral sequences. Nucleic Acids Res. 2016;44(D1):D286–93.

19. Bolger AM, Lohse M, Usadel B. Trimmomatic: A flexible trimmer for Illumina sequence data. Bioinformatics. 2014;30(15):2114–20.

20. Bengtsson-Palme J, Hartmann M, Eriksson K, Pal C, Thorell K, Larsson J, et al. Metaxa2: Improved Identification and Taxonomic Classification of Small and Large Subunit rRNA in Metagenomic Data. Vol. 15, Molecular Ecology Resources. 2015.

21. Langmead B, Salzberg SL. Fast gapped-read alignment with Bowtie 2. 2012;9(4):357–60.

22. Li H, Handsaker B, Wysoker A, Fennell T, Ruan J, Homer N, et al. The Sequence Alignment/Map format and SAMtools. Bioinformatics [Internet]. 2009 Aug 15;25(16):2078–9. Available from: http://dx.doi.org/10.1093/bioinformatics/btp352

23. Li H. A statistical framework for SNP calling, mutation discovery, association mapping and population genetical parameter estimation from sequencing data. Bioinformatics [Internet]. 2011 Nov 1;27(21):2987–93. Available from: http://dx.doi.org/10.1093/bioinformatics/btr509

24. Barnett DW, Garrison EK, Quinlan AR, Strmberg MP, Marth GT. Bamtools: A C++ API and toolkit for analyzing and managing BAM files. Bioinformatics. 2011;27(12):1691–2.

25. Peng Y, Leung HCM, Yiu SM, Chin FYL. Meta-IDBA: a de Novo assembler for metagenomic data. Bioinformatics [Internet]. Oxford University Press; 2011 Jul 1;27(13):i94–101. Available from: https://pubmed.ncbi.nlm.nih.gov/21685107

26. NCBI NR-database. Version 2017-03-27. ftp://ftp.ncbi.nih.gov/blast/db/FASTA/nr.gz. Accessed November 2017

27. Protein accession to EggNOG mapping file (MEGAN6). http://ab.inf.uni-tuebingen.de/data/software/megan6/download/ acc2eggnog-Oct2016X.abin.zip . Accessed 29 November 2018.

28. Protein accession to NCBI-taxonomy mapping file. http://ab.inf.uni-tuebingen.de/data/software/megan6/download/ prot_acc2tax-Nov2018X1.abin.zip. Accessed 29 November 2018.

29. FastX-Toolkit. http://hannonlab.cshl.edu/fastx_toolkit/. Accessed 08 January 2019
